# Uric Acid, Hypertensive Phenotypes, and Organ Damage: Data from the Pamela Study

**DOI:** 10.1007/s11906-022-01174-9

**Published:** 2022-01-25

**Authors:** Guido Grassi, Jennifer Vanoli, Rita Facchetti, Giuseppe Mancia

**Affiliations:** 1 Department of Medicine and Surgery, Clinica Medica, University of Milano-Bicocca, Milan, Italy; 2 University of Milano-Bicocca, Milan, Italy

**Keywords:** Uric acid, Ambulatory blood pressure, Home blood pressure, Office blood pressure, Blood pressure variability, Organ damage, Cardiovascular risk

## Abstract

**Purpose of Review:**

To examine published and unpublished data collected in the context of the Pressioni Arteriose Monitorate E Loro Associazioni (PAMELA) study on the relationships between serum uric acid (SUA), office and out-of-office blood pressure (BP), and organ damage.

**Recent Findings:**

SUA values were directly and significantly related to a large number of covariates that participate at cardiovascular risk determination, such as blood glucose, total serum cholesterol, serum triglycerides, body mass index, and serum creatinine. Additional variables included echocardiographically-determined left ventricular mass index and BP values, the latter not just when measured in the office but also when evaluated at home or over the 24-h period. White-coat hypertension and masked hypertension were characterized, as sustained hypertension, by a significant increase in SUA levels, which were also directly related to different indices of 24-h BP variability. No substantial difference in SUA levels was found when data were analyzed according to the dipping or non-dipping nocturnal BP profile.

**Summary:**

Data collected in the frame of the PAMELA study document the presence of a close relationship between SUA levels and BP values independently on the hypertensive phenotype patterns of BP increase (office, 24 h, or both) and nighttime BP profile. They also document the increase in SUA as a potential factor favoring the occurrence of new hypertension and new left ventricular hypertrophy.

## Introduction

Serum uric acid (SUA) represents the end-product of purine metabolism, and its over-production and reduced renal excretion are responsible for development of a hyperuricemic state in humans [1]. Several studies in the literature report the relationships between SUA levels and well-recognized cardiovascular risk factors, including high BP [2–8], showing that SUA is capable to predict, independent of other confounders, fatal and non-fatal cardiovascular events in hypertension (HT), in diabetes mellitus, and in healthy middle-aged and elderly individuals [9–14]. This has led the 2018 guidelines for the management of essential hypertension issued by the European Society of Cardiology/European Society of Hypertension (ESC/ESH) to include SUA among the factors influencing cardiovascular risk in hypertensive patients [15••].

 The aim of the present paper is to critically review published and unpublished original observations on the relevance of SUA in essential hypertension by examining the evidence collected by our group in one of the major observational studies still ongoing in the area of hypertension, i.e., the Pressioni Arteriose Monitorate E Loro Associazioni (PAMELA) study [16•]. The investigation, which recently completed the collection of data after 25 years from the publication of the first survey, represents the epidemiological study in the field of ambulatory and home BP with the longest (a quarter of a century) follow-up never done before. The clinical relevance of the data collected via 24-h and home BP measurement is based on the evidence that these evaluations may predict cardiovascular events and organ damage development in a manner more accurate and sensitive than office BP [17••].

In the present review, we will examine data on the association between SUA and hypertension considering not only office, but also out-of-office BP values (24-h ambulatory or home), and in particular the relationships with different BP phenotypes as well as with different indices of BP variability. The relationships between SUA profiles and organ damage are finally examined, again taking into account mainly the data collected in the frame of the PAMELA study.

## An Outlook on the PAMELA Study

At the beginning of the Nineties, in the past century, the PAMELA study was carried out on a sample of 3200 individuals randomly selected to be representative of the population of Monza, a city located in an urban area with high population density in Lombardy, northern Italy [16•]. The study protocol was approved by the ethic committee of one of the Institutions involved. A total of 2051 persons joined to participate in the research (participation rate 64%). Study participants were evaluated in the outpatient clinic of the San Gerardo Hospital of Monza, and full medical history, blood and urine samples, physical examination, and three sphygmomanometric (office) systolic and diastolic BP measurements in the sitting position were collected. Body weight and height were also recorded. Blood sampling allowed assay of several laboratory variables including plasma glucose, triglycerides, HDL, LDL and total cholesterol, creatinine, electrolytes, and serum uric acid. Other measurements included echocardiographic evaluation of left ventricular function and structure, with a precise determination of left ventricular ejection fraction and wall thickness, and of the diameter of the left atrium [16•]. All participants also underwent ambulatory BP monitoring, with the device set to obtain automated BP and heart rate oscillometric readings every 20 min over the 24-h period [16•]. During the monitoring period, participants were instructed to perform their normal activities, to hold the arm still at time of BP reading, and to standardize the time of activities, particularly in relation to sleeptime at night. Ambulatory BP recordings were analyzed to obtain 24-h, daytime, and night-time average systolic/diastolic values and heart rate, after editing for artifacts, based on preselected criteria. Home blood pressure was taken by each participant via a validated semiautomatic BP measuring device.

 According to office and out-of-office BP values, participants were divided into 4 groups characterized by different BP phenotypes, i.e., (1) true normotensive subjects with normal office (140/90 < mmHg) and 24-h (< 125/79 mmHg) BP, (2) white-coat hypertensive subjects displaying elevated office but normal ambulatory BP, (3) masked hypertensive individuals with normal office but elevated ambulatory BP, and (4) sustained hypertensives with elevated office and ambulatory BP [15••, 16•]. Along with absolute BP values in the PAMELA study, data were also collected on BP variability, namely, the oscillations in BP occurring during the 24-h period. To this aim, the standard deviation of 24-h, daytime, and nighttime BP values were calculated together with the day-night BP difference and the residual or erratic BP variability (Fourier spectral analysis) [18].

## Uric Acid Profile in the PAMELA Study

In the PAMELA study, population mean values of SUA were 4.94 ± 1.34 mg/dl (mean ± standard deviation), with a near normal Gaussian distribution, which means that the values displayed a probability distribution that is symmetric about the mean, and shows that data near the mean were more frequent in occurrence than data far from the mean [19]. This average value and value distribution were recently confirmed by the results of one of the largest surveys assessing the clinical relevance of SUA in relation to cardiovascular risk, the Uric Acid Right for Heart Health (URRAH) study, sponsored by the Italian Society of Hypertension [20•].

**Fig. 1:**
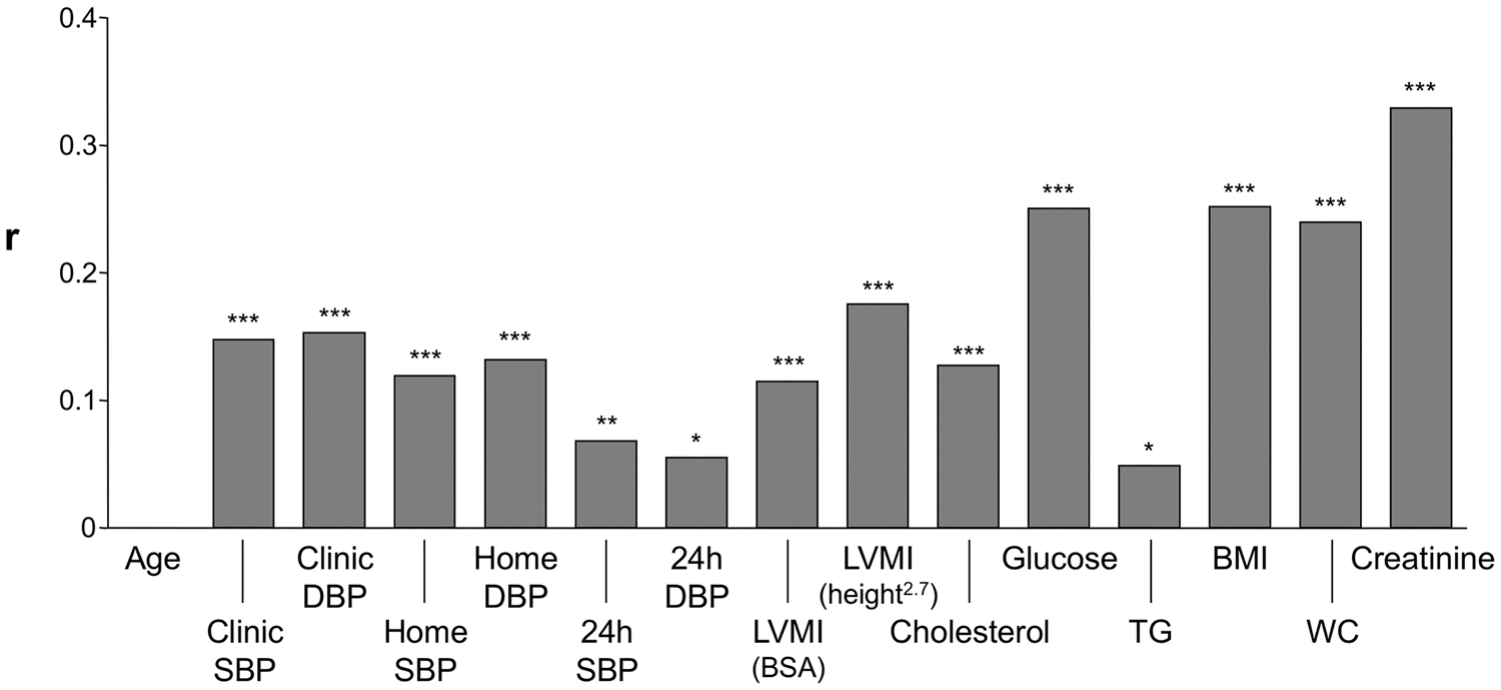
Correlation coefficients (*r*) of the relationships between serum uric acid (SUA) and different anthropometric, hemodynamic, and metabolic variables collected in the PAMELA Study. Asterisks refer to the level of statistical significance (*P < 0.05, **P < 0.01) of the various correlations. DBP diastolic blood pressure, SBP systolic blood pressure, LVMI left ventricular mass index, BSA body surface area, TG triglycerides, BMI body mass index, WC waist circumference. Figure drawn from original data shown as table in Ref 19

One of the first analyses of the PAMELA study focused on the relationships between SUA and BP. As mentioned above, the methodology to assess BP included a variety of techniques. This allowed us to obtain important information regarding the relationships between SUA and BP phenotypes, and thus on the impact of SUA on cardiovascular risk. As shown in Fig. 1, SUA was significantly and directly related to several variables that contribute to cardiovascular risk determination [19]. These included blood glucose, total serum cholesterol, serum triglycerides, and serum creatinine, as well as body mass index, echocardiographic left ventricular mass, and BP evaluated by both office measurements and home or 24-h readings. These findings confirm the notion that elevated SUA levels mirror the presence of an altered metabolic risk factor profile, detection of structural alterations of the heart, and elevation in clinic and ambulatory BP. Thus, an increased SUA is a sensitive marker of a variety of clinical conditions which have an adverse impact on cardiovascular risk profile. Another sub-analysis of the data collected in the PAMELA study was related to the prognostic value of SUA in determining future development of hypertension [19]. This was done in 2045 patients who were longitudinally evaluated, after the first examination, during a 10-year follow-up, examining not only office but also out-of-office BP data. Results show that compared to the patients who did not develop high BP during the follow-up, those developing hypertension displayed greater baseline values of age, body mass index, glucose, and SUA. These findings demonstrate the relevance of metabolic factors, and in particular elevated SUA levels, for the development of a new hypertensive state, detected via office, home, or ambulatory BP measurements.

## Uric Acid and BP Patterns

**Fig. 2:**
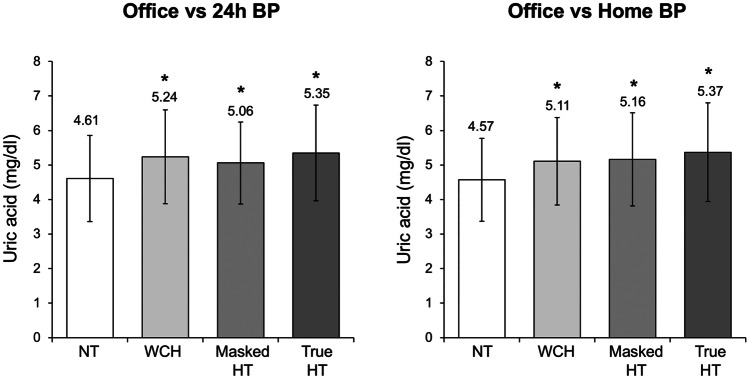
Bar graphs showing serum uric acid values in the normotensive subjects (NT) and in white coat (WCH), masked (masked HT), and sustained (true HT) hypertensive patients recruited in the PAMELA study. Data have been analyzed considering office vs 24-h blood pressure (BP, left panel) and office vs home blood pressure (BP, right panel). Asterisks (*P < 0.05) refer to the statistical significance of serum uric acid values in the 3 above mentioned hypertensive populations compared to normotensive controls (NT). Data are shown as means ± standard deviation

A further analysis of the PAMELA data focused on the relationships between SUA and 24-h BP patterns. Specifically, 932 patients were identified as true normotensives based, as previously mentioned, on the normal BP values detected by sphygmomanometric (office) and 24-h (ambulatory) BP measurements. Of these, 285 had white-coat hypertension, while 225 had masked hypertension. A total of 611 patients had sustained hypertension with elevation of both clinic and ambulatory BPs. Compared to the normotensive individuals, patients with “white-coat hypertension,” “masked hypertension,” and “sustained hypertension” were older and displayed greater body mass index, serum total cholesterol, triglycerides, and blood glucose. This was true whether BP normality and elevation were identified by office versus out-of office criteria. Similarly, as shown in Fig. 2, mean SUA levels were greater in white-coat hypertension, masked hypertension, and sustained hypertension compared to the normotensive state, respectively in both conditions based on data analysis office versus 24 h and office versus home. There was a tendency which remained significant even after data adjustments for confounders (age, sex, smoking habit, body mass index, serum creatinine, blood glucose and lipid variables, and left ventricular mass index) for SUA levels to be more elevated in sustained hypertension rather than in the other two hypertensive phenotypes. SUA levels were also directly correlated to the number of elevated BP values. The mean of SUA was significantly greater in patients with at least one elevated BP value compared to normotensives, with a progressive increase in SUA levels as the number of elevated BP values increased.

Taken together, these findings are consistent with the evidence, provided by the PAMELA study as well as by other epidemiological surveys, that white-coat hypertension and masked hypertension cannot be regarded as innocent conditions. The elevations in SUA levels being are a further demonstration that these two phenotypes have a pathophysiological background more similar to sustained hypertension than to the normotensive state. A further conclusion from these findings is that elevated BP, whether detected at home, during the 24 h or at a hospital visit, is associated with increased circulating plasma uric acid levels.

 A further evaluation of the relationship between SUA and BP was focused on the SUA in patients with “dipping” or “non-dipping” BP patterns during the nighttime period, phenotypes which have been shown to have different cardiovascular risk profiles [15••]. “Non-dipper” hypertensives, i.e., patients showing an attenuated or absent BP decrease during night, display more pronounced cardiac and extra-cardiac organ damage compared with subjects characterized by a normal nocturnal BP profile. They also display an increased risk of developing fatal and non-fatal cardiovascular events [15••]. However, our data analysis failed to show any substantial differences of SUA values between the two conditions, a finding suggesting that uric acid is not involved in the worse cardiovascular risk profile of the non-dipping phenotype. Similar results were obtained when the SUA profile was assessed taking into account the reverse or inverted dipping profile, i.e., the hypertensive phenotype characterized by higher nocturnal BP compared with daytime BP values, responsible for an elevated cardiovascular risk profile [15••].

**Fig. 3:**
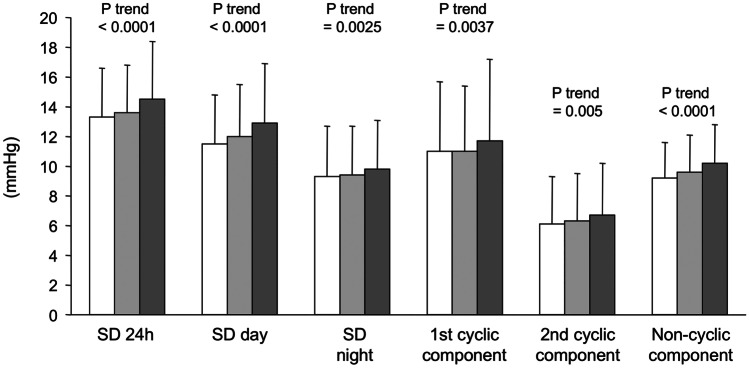
Values of 24 h, daytime and nighttime systolic blood pressure variability, expressed as standard deviation (SD) and of the first, the second, and the non-cyclic components of systolic BP variability in relation to tertiles of SUA in the PAMELA population. *P* values for trend are shown for each variable. SUA values: First tertile (open columns): ≤ 4.2 mg/dl, second tertile (grey columns): 4.2–5.4 mg/dl and third tertile (black columns) > 5.4 mg/dl. Data are shown as means ± standard deviation

Finally, an additional analysis of the SUA-BP relationships in the PAMELA study was focused on the possible interactions of this metabolic parameter with BP variability. The issue is of particular interest given the evidence that cardiovascular prognosis is related not only to the absolute BP load during the 24 h period but also, and probably to a greater extent, to the BP oscillations occurring during the daytime and the nighttime period [15••]. In particular, data from the PAMELA study have shown that BP variability (specifically residual variability, which accounts for about 50% of overall variability [21]) is significantly and directly related to left ventricular mass index, suggesting that large BP oscillations during the 24 h may be responsible for the structural alterations of the left ventricle, independently of the 24 h BP load [21]. For this analysis, we divided the population into three tertiles according to SUA levels, first tertile ≤ 4.2 mg/dl, second tertile 4.2–5.4 mg/dl, and third tertile > 5.4 mg/dl (Fig. 3). Analysis of the data relating SUA with BP variability shows that in the population examined, the increase in SUA was accompanied by a progressive increase in BP variability. This was specifically the case when different measures of BP variability (24-h standard deviation, day-time standard deviation, first cyclic component, and the residual uncyclic component) were taken into account (Fig. 3).

## Uric Acid and Cardiac Damage

In the past few years, growing interest has been focused on the hypothesis that SUA may exert direct and indirect adverse effects on cardiac function and structure. Evidence has been collected, in the frame of the already mentioned URRAH study, which includes data collected in about 20 centers of excellence belonging to the Italian Society of Hypertension for a total of more than 21,000 subjects, that SUA values > 5.34 mg/dl are strong independent predictors of future development of congestive heart failure, even when data are adjusted for various confounders [22•]. These findings therefore extend information collected in previous studies on SUA and heart failure in a very large number of subjects, and the conclusion that the involvement of SUA in heart failure risk stratification is directly related to its circulating plasma levels.

**Fig. 4:**
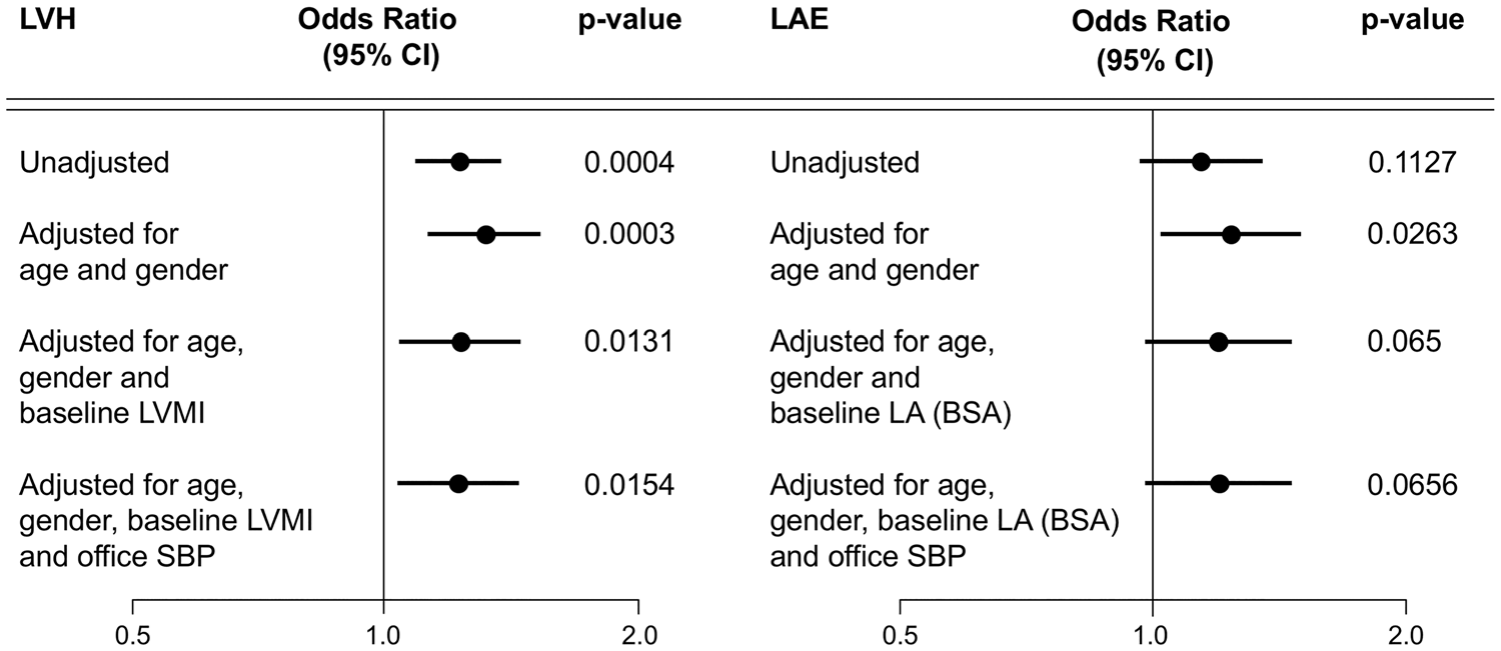
Odd ratio (OR) and 95% confidence interval (95% CI) of developing new left ventricular hypertrophy (LVH, echocardiographic evaluation, left panel) and new left atrial enlargement (LAE, echocardiographic evaluation, right panel) associated with a 1-mg/dl increase of serum uric acid. Data are shown as unadjusted and adjusted values for different confounders. LVMI left ventricular mass index, LA left atrium diameter, BSA body surface area, SBP systolic blood pressure

The relevance of SUA to the development and progression of the structural alterations of heart was confirmed by the evidence that SUA levels predicted the future development of left ventricular hypertrophy in 960 patients followed for a decade in the PAMELA study [23•]. The association was significant after data adjustment for confounders, including age, gender, metabolic risk factors, entry left ventricular mass index, BP, and antihypertensive drugs. The independent predictive role of SUA was major, as each 1 mg/dl SUA increase after adjustment was associated with a 26% increased risk of left ventricular hypertrophy (Fig. 4, left panel). This unfavorable trend was even more evident when left ventricular hypertrophy incidence in the previously mentioned highest SUA tertile was compared to the lowest tertile, as patients in the former group had a 96% greater risk of developing structural alterations of the left ventricle compared to their counterparts.

Two final sets of original data, again from the PAMELA population, are worthy of mention. First, when we analyzed the SUA data in relation to the new development of left atrial enlargement, as assessed by serial echocardiographic evaluation of left atrial diameters during a 10-year follow-up period. We were unable to find a significant relationship between SUA values and left atrial enlargement, as each 1 mg/dl SUA increase after adjustment for age, gender, and baseline left atrial diameter was accompanied by a non-significant increase in the risk of developing this structural atrial alteration (Fig. 4, right panel). This may suggest that the effects of SUA on the cardiovascular system are more prompted to interfere with processes promoting cardiac hypertrophy, e.g., activation of smooth muscle cell proliferation, stimulation of inflammatory mediators, and of mitogen-activated protein kinases, rather than with factors favoring the cardiac remodeling process, e.g., cardiac wall stress, modification of distending or deforming myocardial process, myocite enlargement, and myocardial fibrosis.

Second, the relationships we observed between SUA, BP profile, and patterns of target organ damage are valid for the uncomplicated hypertensive state. When we assessed these variables in hypertensive patients with comorbidities, many of the observed relationships had weaker or no statistical significance. This was particularly true for obesity, which in the PAMELA study was detectable in about 14% of the population screened. The obese individuals characterized by body mass index values > 30 kg/m^2^ and waist circumference > 102 cm in males and 88 in females displayed significantly greater values of SUA compared to the normal weight individuals, and there was a significant relationship between SUA and waist circumference, taken as a variable capable to provide sensitive information on fat accumulation. However, when we evaluated the ability of SUA in the obese individuals to predict the development of future hypertension, no significant relationship was found, even when the data were not adjusted for confounders. Similar results were found for the possible prognostic value of SUA for cardiovascular events. These findings may suggest that the impact of SUA as prognostic marker is specific for the hypertensive state. It is likely hypothesized that human obesity and other factors, such as insulin sensitivity, glycemic imbalance, oxidative stress, endothelial dysfunction, impairment of autonomic cardiovascular control, sleep apnea, may have greater prognostic relevance than SUA [24].

## Conclusions

The data discussed in this paper strengthen the importance of SUA as an independent risk factor for the development of hypertension and hypertension-related cardiac organ damage. The close relationship between SUA and BP has therapeutic relevance, given the evidence that elevated SUA levels may favor the development of resistance to antihypertensive drug treatment [25•] and the association of urate lowering therapy with antihypertensive effects [26•]. Ongoing studies will provide in a near future insights on the pathophysiological mechanisms linking SUA, elevated BP, and antihypertensive drug treatment.
